# Supplementary material for: Content-rich biological network constructed by mining PubMed abstracts
Source: BMC Bioinformatics. 2004 Oct 8;5:147. doi: 10.1186/1471-2105-5-147 (PMC528731; doi:10.1186/1471-2105-5-147)
Supplement: Additional File 2 — The original results of the above study (non-essential files are deleted to keep the file size under the limit set by BMC bioinformatics). [file 1471-2105-5-147-S2.bz2 › chilibotAdditionalFile2/dip10/296ID7486683E1287/html/GRB2_IRS1.html]

 


 **GRB2** and **IRS1** 
  
Found 180 abstracts in PubMed, retrieved 10.  
 

 What does Google say? 
 PDF only 
| .edu only 

---

**Interactive relationship** (e.g. stimulation, inhibition, etc)

**Inhibitory relationship**- With regard to the large effect on MAPK phosphorylation, we found that PTP1B overexpression led to the impaired phosphorylation of both IRS 1  [ **IRS1** ]  and Shc, resulting in a decrease in their association with  **Grb2** .  Ref: 12446583 Endocrinology, 2002
**Neutral relationship**- Signalling by the insulin receptor substrate IRS  [ **IRS1** ]  proteins is critically dependent on the tyrosine phosphorylation of specific binding sites that recruit Src homology 2 SH2 domain containing proteins, such as the p85 subunit of phosphoinositide 3 kinase PI 3 kinase, the tyrosine phosphatase SHP 2 and the adapter protein  **Grb2** .  Ref: 12220227 Biochem J, 2002

**Non-interactive relationship** (e.g. studied together, co-existance, homology, etc.)

- TGF beta1 affected IGF I stimulated insulin receptor substrate 1  [ **IRS1** ]  IRS 1  [ **IRS1** ]  tyrosine phosphorylation and its association with  **Grb2**  protein.  Ref: 12837295 Exp Cell Res, 2003
- Conversely, TGF beta1 did not alter IGF I stimulated IRS 1  [ **IRS1** ]  associated phosphatidylinositol 3 kinase activity, IGF I induced tyrosine phosphorylation of Shc, and its binding to  **Grb2** .  Ref: 12837295 Exp Cell Res, 2003
- We observed association between BCR ABL IRS 1  [ **IRS1** ] , IRS 1  [ **IRS1** ]  phosphoinositide 3 kinase PI3 kinase, and IRS 1  [ **IRS1** ]   **Grb2**  in the K562 cell line.  Ref: 12560071 FEBS Lett, 2003
